# Comparison of metabolic syndrome prevalence using four different definitions – a population-based study in Finland

**DOI:** 10.1186/s13690-021-00749-3

**Published:** 2021-12-23

**Authors:** Elsi Haverinen, Laura Paalanen, Luigi Palmieri, Alicia Padron-Monedero, Isabel Noguer-Zambrano, Rodrigo Sarmiento Suárez, Hanna Tolonen, for the Joint Action on Health Information (InfAct)

**Affiliations:** 1 Department of Public Health and Welfare, Finnish Institute for Health and Welfare (THL), Mannerheimintie 166, 00271 Helsinki, Finland; 2 Department of Cardiovascular, Endocrine-metabolic Diseases and Aging Istituto Superiore di Sanità (ISS), Via Giano della Bella, 34, 00161 Rome, Italy; 3 National School of Public Health, Instituto de Salud Carlos III, C/ Monforte de Lemos 5, Madrid, Spain

**Keywords:** Metabolic syndrome, Population health survey, Health examination survey, Indicators, Standardization, Diabetes

## Abstract

**Background:**

Metabolic syndrome (MetS) is a public health problem in Europe, affecting all age groups. Several MetS definitions are available. The aim of this study was to compare four different MetS definitions in the Finnish adult population, to assess their agreement and to evaluate the impact of the choice of the definition on the prevalence of MetS.

**Methods:**

Data from FinHealth 2017, a cross-sectional national population health survey, focusing on adults aged 25 years or older were used in the analysis (*n*=5687). Measured data on anthropometrics, blood pressure and biomarkers together with questionnaire data were used to classify the participants into the MetS categories according to the four definitions. The definitions chosen for the comparison were those by the World Health Organization (WHO) (1998), National Cholesterol Education Program Adult Treatment Panel III (NCEP-ATP III) (2004), International Diabetes Federation (IDF) (2005), and Joint Interim Statement (JIS) (2009).

**Results:**

The four MetS definitions resulted in substantially different MetS prevalence: 17.7% by WHO, 33.3% by NCEP-ATP III, 41.5% by IDF, and 43.0% by JIS. Regardless of the definition used, the prevalence of MetS increased with age. The prevalence of the different components varied between the definitions, depending on the different cut-off points adopted. Out of all participants, only 13.6% were identified to have MetS according to all four definitions. Agreement between participants recognised by different MetS definitions, estimated through kappa coefficients, was almost perfect for IDF vs. JIS (0.97), strong for JIS vs. NCEP-ATP III (0.80), moderate for IDF vs. NCEP-ATP III (0.76) and weak for WHO vs. NCEP-ATP III (0.42), WHO vs. IDF (0.41) and WHO vs. JIS (0.40).

**Conclusions:**

Differences between observed prevalence of MetS in Finnish men and women using different MetS definitions were large. For cross-country comparisons, as well as for trend analyses within a country, it is essential to use the same MetS definition to avoid discrepancies in classification due to differences in used definitions.

**Supplementary Information:**

The online version contains supplementary material available at 10.1186/s13690-021-00749-3.

## Background

Metabolic syndrome (MetS) is an increasing public health concern globally [1–3]. MetS is a cluster of risk factors, comprising obesity, elevated blood pressure and distorted lipid- and glucose metabolism [4]. The risk of coronary heart disease, stroke, non-alcoholic fatty liver disease, diabetes mellitus type 2 (T2DM), other forms of cardiovascular diseases, and all-cause mortality is directly linked to MetS [5–10]. While the pathogenic mechanisms remain under debate, it has been suggested that chronic inflammation might be an underlying mechanism in the development of MetS [11]. Furthermore, this hypothesis is in line with the concept of metaflammation (metabolic inflammation resulting from nutrient excess) that contributes to the pathogenesis of insulin resistance and cardiometabolic diseases [12]. The increased global MetS prevalence may be partly explained by increased sedentary lifestyle [13] as well as unfavorable diet [14] and possible modification of several lifestyle behaviours that have additionally been associated with a lower frequency of MetS [15]. Environmental chemical exposure has also been suggested to play a role in the increasing global MetS trend [16, 17].

The prevalence of MetS differs according to the definition, geographic location and population examined, however the prevalence has been estimated to be somewhere between 13 and 36% among the European populations studied [18–21]. Although the risk of MetS has been observed to be higher at older age [21–23], the evidence also suggests a risk of MetS already among younger age groups [24, 25]. However, studies on MetS among younger adults are scarce. Furthermore, development of MetS has been shown to have some gender differences according to age, men having higher prevalence at younger age compared to women, although the gender difference reverses among older age groups [26].

Currently, several well-known and accepted MetS definitions are used in clinical and research fields including the same components (obesity, glucose and lipid metabolism and blood pressure), however the definitions emphasize different components and use different cut-off points. E.g. the definition by the World Health Organization (WHO) [4] emphasizes insulin resistance, where the International Diabetes Federation (IDF) [27] has a prerequisite of central obesity. Due to the variation, these definitions may identify partly different individuals [19]. Qiao et al. (2016) discovered in their study that of 2116 men who had MetS by any of the three included definitions (WHO, the National Cholesterol Education Program (NCEP) and IDF), only 33.4% met the criteria of all three [19].

This paper was prepared under the framework of the Joint Action on Health Information (InfAct) which builds towards a sustainable and solid infrastructure on EU health information. One of the goals of InfAct is to support health information interoperability which also includes standardized definitions for used health indicators. The objective of the present study was to compare four different MetS definitions: WHO 1998 [4]; National Cholesterol Education Program Adult Treatment Panel (NCEP-ATP III) 2004 [28]; IDF 2005 [27] & Joint Interim Statement (JIS) 2009 [29] among adults aged 25 years and older using data from Finland to demonstrate differences using real data, to assess their agreement and to evaluate the impact of the choice of the definition on the prevalence of MetS. Additionally, we compared the four definitions by gender and age.

## Methods

### Definitions included in the comparison

Four established and widely used definitions were chosen for the comparison. These were the definitions of the WHO [4], NCEP-ATP III [28], IDF [27] and JIS [29] (Table 1). In this study, the WHO’s definition was modified to exclude impaired glucose tolerance (IGT), Homeostatic Model Assessment of Insulin Resistance (HOMA-IR) and microalbuminuria as these measurements were not available from the FinHealth 2017 survey data, and additionally the blood pressure cut-off was set at 140/90 mmHg in accordance to the previous literature [19, 30, 31] and WHO’s clinical guidelines for blood pressure [32]. For NCEP-ATP III, the definition was modified to include the lower cut-off points for fasting plasma glucose based on the report by the American Heart Association/National Heart, Lung, and Blood Institute/American Diabetes Association’s recommendations [33, 28]. The IDF and JIS definitions were modified in respect to triglycerides and high-density lipoprotein cholesterol (HDL-C) criteria. Information on specific treatment for these lipid abnormalities was not available and thus could not be included in the criteria applied in this study. Waist circumference criteria for IDF and JIS followed the country and population specific Europid cut-off points [27, 29].

**Table 1:** Diagnostic criteria used for metabolic syndrome by four metabolic syndrome definitions

Clinical measure	WHO (1998)	NCEP-ATP III (2004)	IDF (2005)	JIS (2009)
**Criteria needed for definition**	**insulin resistance + at least two further criteria needed**	**At least three of the following**	**Central obesity (WC) + at least two of the following**	**At least three of the following**
**Central obesity**	M: WHR > 0.90 F: WHR > 0.85 and/or BMI > 30 kg/m^2^	M: WC ≥ 102 cm F: WC ≥ 88 cm	Increased WC (population specific) Europids: M: ≥ 94 cm F: ≥ 80 cm	Increased WC (population and country specific) Europids: M: ≥ 94 cm F: ≥ 80 cm
**Lipid metabolism**	TG ≥ 150 mg/dl (1.7 mmol/l) and/ or M: HDL-C < 35 mg/dl (0.9 mmol/l) F: HDL-C < 39 mg/dl (1.0 mmol/l)	TG ≥ 150 mg/dl (1.7 mmol/l)	TG ≥ 150 mg/dl (1.7 mmol/l)	TG ≥ 150 mg/dl (1.7 mmol/l)
		M: HDL-C < 40 mg/dl (1.03 mmol/l) F: HDL-C < 50 mg/dl (1.3 mmol/l)	M: HDL-C < 40 mg/dl (1.03 mmol/l) F: HDL-C < 50 mg/dl (1.3 mmol/l)	M: HDL-C < 40 mg/dl (1.03 mmol/l) F: HDL-C < 50 mg/dl (1.3 mmol/l)
**Blood pressure (mmHg)**	≥140/90 or medication	≥130/85	≥130/85 or medication	≥130/85 or medication
**Glucose metabolism/insulin resistance**	FPG ≥ 110 mg/dl (6.1 mmol/l) (indicating IFG) or T2DM diagnosis	FPG >100 mg/dl (5.6 mmol/l) or medication	FPG ≥ 100 mg/dl (5.6 mmol/l) or T2DM diagnosis	FPG ≥ 100 mg/dl (5.6 mmol/l) or medication

WHO= World Health Organization, NCEP-ATP III = National Cholesterol Education Program Adult Treatment Panel III, IDF= International Diabetes Federation, JIS= Joint Interim Statement, M= male, F= female, IFG=impaired fasting glucose, FPG= fasting plasma glucose, T2DM= type 2 diabetes mellitus, WC= waist circumference, WHR= waist to hip ratio, BMI= body mass index, TG= triglycerides, HDL-C= high-density lipoprotein cholesterol

### Study population

The total sample size of the FinHealth 2017 Study was 10,007 persons aged 18 years and older. The survey was conducted as a cross-sectional national population-based health examination survey during the first six months of 2017 in the mainland Finland. The study was based on a nationally representative sample, using two-stage random sampling. The data were collected through self-administered questionnaires, health interviews and standardized health examination measurements. The health examination included anthropometric measurements, blood pressure measurement and blood sampling. The methods, including a detailed description of the sampling design, have been published previously [34].

The analyses of the current study were performed among 5687 FinHealth 2017 survey participants. The final study population included 2621 (46%) men and 3066 (54%) women aged at least 25 years for whom information for all components of the included MetS definitions was available (Table 2). The participation rate was higher among women than men. The highest participation rate was reached in the age group of 60-79 years and the lowest participation rate appeared in the youngest age group of under thirty years [34]. The mean age for men was 54.4 (range 25.0-93.4) and for women 55.2 (range 25.0-98.7) years.

**Table 2:** Baseline characteristics of the study participants

	Men (*n*=2621)		Women (*n*=3066)		Total (*n*=5687)	
	mean	SD	mean	SD	mean	SD
Age	54.4		55.2		54.8	
BMI (kg/m^2^)	27.6	±4.4	27.4	±5.5	27.5	±5.0
WC (cm)	99.1	±12.9	89.6	±14.1	94.0	±14.4
SBP (mmHg)^a^	135.9	±17.9	132.8	±20.4	134.2	±19.4
DBP (mmHg)^a^	81.2	±11.2	77.8	±10.6	79.4	±11.0
HDL-C (mmol/l)	1.3	±0.3	1.6	±0.4	1.5	±0.4
TGs (mmol/l)	1.5	±1.1	1.3	±0.7	1.4	±0.9
FPG (mmol/l)^b^	6.0	±1.4	5.6	±1.1	5.8	±1.3

SD= standard deviation, BMI= body mass index, WC= waist circumference, SBP= systolic blood pressure, mmHg= millimeters of mercury, DBP= diastolic blood pressure, HDL-C= high density lipoprotein cholesterol, TGs= triglycerides, FPG= fasting plasma glucose

^a^mean value of two measurements

^b^minimum of 4 h fasting plasma glucose

### Data collection

During the health examination survey, weight, height and blood pressure as well as waist and hip circumferences were measured, and blood samples were drawn according to the European Health Examination survey protocols [35]. Weight (in kg) was measured in light clothing with Tanita DC-430-MA body composition analyser. Height was measured with a portable Seca 213 stadiometer and applied in the analyses in meters as it was used for body mass index (BMI) calculation. BMI was calculated as weight divided by squared height. Waist and hip circumferences were measured on bare skin or light underwear using a plastic measuring tape. Waist circumference was measured midway between the lower ribs margin and iliac crest, and hip circumference was measured 2.5 cm above the pubic bone. Blood pressure was measured from the right arm, in a sitting position using a mercury sphygmomanometer. Blood pressure was measured three times, and the mean value of the first and second measurement was calculated.

The participants were advised to have minimum of 4 h fasting before the blood sample collection. For this study, the relevant biomarkers determined from the blood samples were fasting plasma glucose, total cholesterol, high density lipoprotein cholesterol and triglycerides. Laboratory analyses were performed in an accredited biochemistry laboratory of the Finnish Institute for Health and Welfare (THL) with a clinical chemistry analyser Architect ci8200 [34]. Cholesterol was assessed using Enzymatic Abbott method (Abel-Kendall verification). Glucose determinations were carried out by enzymatic hexokinase Abbott (NIST SRM 956).

### Statistical analysis

Descriptive analysis included estimations of mean values and standard deviations (SD). The prevalence estimates with 95% confidence intervals (95% CI) were calculated by sex and 10-year age groups using the criteria of each MetS definition. The differences between the definitions were tested using Chi-squared test. Cohen kappa coefficients with 95% CIs were calculated for paired definitions. The prevalences of MetS components were calculated by sex and age separately for each definition. Prevalence ratios (PR) with 95% CIs were calculated by gender and age groups. The youngest age group was set as the unexposed group and the other age groups were compared to it in the calculation of PRs. Statistical analysis was conducted using R studio version 3.6.0.

## Results

The total prevalence of MetS varied between 17.7% and 43.0% when criteria of the four different definitions were used (Table 3, Additional file 1). For both genders, the differences in prevalences between the definitions were significant (*p*<0.001). The observed prevalence rates, from the lowest to the highest, were with WHO (men 22.4% and women 13.7%), NCEP-ATP III (men 35.4%, women 31.6%), IDF (men 45.6%, women 38.0%), and JIS (men 48.1%, women 38.7%). The total prevalence was highest for the JIS and IDF definitions (43.0% for JIS and 41.5% for IDF), while the biggest difference was found between JIS (43.0%) and WHO (17.7%).

**Table 3:** Number of cases and prevalence of MetS with 95% CI according to the different MetS definitions, by gender and age group

			WHO (1998)		NCEP-ATP III (2004)		IDF (2005)		JIS (2009)	
			**n**	**%** **(95% CI)**	**n**	**%** **(95% CI)**	**n**	**%** **(95% CI)**	**n**	**%** **(95% CI)**
**Total**	*n*=5687		**1007**	**17.7 (16.7-18.7)**	**1896**	**33.3 (32.1-34.5)**	**2360**	**41.5 (40.2-42.8)**	**2448**	**43.0 (41.8-44.3)**
**Male**	**Total**	*n*=2621	**588**	**22.4 (20.8-24.0)**	**927**	**35.4 (33.6-37.3)**	**1195**	**45.6 (43.7-47.5)**	**1260**	**48.1 (46.2-50.0)**
	Age group	25-34	15	4.0 (2.5-6.5)	54	14.5 (11.3-18.4)	63	16.9 (13.4-21.0)	72	19.3 (15.6-23.6)
		35-44	30	7.1 (5.0-10.0)	101	24.0 (20.2-28.3)	130	30.9 (26.7-35.4)	141	33.5 (29.1-38.1)
		45-54	101	21.2 (17.8-25.1)	185	38.9 (34.6-43.3)	233	48.9 (44.5-53.4)	247	51.9 (47.4-56.3)
		55-64	179	30.0 (26.5-33.8)	263	44.1 (40.2-48.1)	333	55.9 (51.9-59.8)	346	58.1 (54.0-61.9)
		65-74	188	36.2 (32.2-40.4)	246	47.4 (43.1-51.7)	313	60.3 (56.0-64.4)	320	61.7 (57.4-65.7)
		75+	75	31.8 (26.2-38.0)	78	33.1 (27.4-39.3)	123	52.1 (45.8-58.4)	134	56.8 (50.4-62.9)
**Female**	**Total**	*n*=3066	**419**	**13.7 (12.5-15.0)**	**969**	**31.6 (30.0-33.3)**	**1165**	**38.0 (36.3-39.7)**	**1188**	**38.7 (37.0-40.5)**
	Age group	25-34	4	0.9 (0.4-2.3)	25	5.8 (3.9-8.4)	29	6.7 (4.7-9.4)	31	7.1 (5.1-10.0)
		35-44	13	2.6 (1.5-4.5)	64	13.0 (10.3-16.2)	88	17.8 (14.7-21.5)	88	17.8 (14.7-21.5)
		45-54	48	8.8 (6.7-11.5)	150	27.5 (23.9-31.4)	177	32.4 (28.6-36.5)	179	32.8 (29.0-36.8)
		55-64	89	14.4 (11.9-17.4)	239	38.7 (35.0-42.6)	279	45.2 (41.3-49.2)	285	46.2 (42.3-50.1)
		65-74	142	23.4 (20.2-26.9)	289	47.6 (43.7-51.6)	350	57.7 (53.7-61.5)	358	59.0 (55.0-62.8)
		75+	123	33.3 (28.7-38.3)	202	54.7 (49.6-59.7)	242	65.6 (60.6-70.2)	247	66.9 (62.0-71.5)

Among 5687 participants, only 775 (13.6%) were identified to have MetS according to all four definitions. Paired comparison of the definitions revealed high variation in participants recognised by the definitions (Table 4). The highest agreement was observed between IDF and JIS (k=0.97, 95% CI 0.96-0.97). Strong agreement was seen for JIS and NCEP-ATP III (k=0.80, 95% CI 0.78-0.81) and moderate for IDF and NCEP-ATP III (k=0.76, 95% CI 0.74-0.78). Weak agreement was detected for WHO and NCEP-ATP III (k=0.42, 95% CI 0.39-0.44), WHO and IDF (k=0.41, 95% CI 0.39-0.43), and WHO and JIS (k=0.40, 95% CI 0.38-0.42).

**Table 4:** Paired comparisons of MetS definitions and Cohen kappa correlation coefficients

Paired definitions	Recognised by both definitions n, (%)	Not recognised by either definition n, (%)	Recognised by the indicated criteria only n, (%)	Recognised by the indicated criteria only n, (%)	Kappa coefficient	95% CI
			**WHO**	**NCEP-ATP III**		
**WHO vs. NCEP-ATP III**	800 (14.1)	3584 (63.0)	207 (3.6)	1096 (19.3)	0.42	0.39-0.44
			**WHO**	**IDF**		
**WHO vs. IDF**	933 (16.4)	3253 (57.2)	74 (1.3)	1427 (25.1)	0.41	0.39-0.43
			**WHO**	**JIS**		
**WHO vs. JIS**	956 (16.8)	3188 (56.1)	51 (0.9)	1492 (26.2)	0.40	0.38-0.42
			**IDF**	**NCEP-ATP III**		
**IDF vs. NCEP-ATP III**	1807 (31.8)	3238 (56.9)	553 (9.7)	89 (1.6)	0.76	0.74-0.78
			**JIS**	**NCEP-ATP III**		
**JIS vs. NCEP-ATP III**	1896 (33.3)	3239 (57.0)	552 (9.7)	0 (0)	0.80	0.78-0.81
			**IDF**	**JIS**		
**IDF vs. JIS**	2357 (41.4)	3236 (56.9)	3 (0.05)	91 (1.6)	0.97	0.96-0.97

### Prevalence by age groups

Regardless of the used definition, the pattern of MetS prevalence by age was similar. Although the differences between all age groups were not statistically significant, the results suggested an increasing prevalence of MetS for men by age, except for the oldest age group of 75+ years, whereas for women the prevalence tended to increase throughout the age groups (Tables 3 and 5, Additional file 1).

The variation between the age groups was smallest using the WHO definition (men: from 4.0 to 36.2%, women: from 0.9 to 33.3%). The patterns for IDF and JIS were very similar with each other. The prevalence range of MetS using both the IDF and JIS definition was wide across different age groups (IDF men: from 16.9 to 60.3%, women: from 6.7 to 65.6%; JIS men: from 19.3 to 61.7%, women: from 7.1 to 66.9%).

**Table 5:** The prevalence of MetS by age group with four definitions, the prevalence ratios (PR) and 95% CIs for age groups

**Male**												
	**WHO**			**NCEP-ATP III**			**IDF**			**JIS**		
**Age groups**	**%**	**PR**	**95% CI**	**%**	**PR**	**95% CI**	**%**	**PR**	**95% CI**	**%**	**PR**	**95% CI**
**25-34**	4.0	1.0		14.5	1.0		16.9	1.0		19.3	1.0	
**35-44**	7.1	1.8	1.0-3.2	24.0	1.7	1.2-2.2	30.9	1.8	1.4-2.4	33.5	1.7	1.4-2.2
**45-54**	21.2	5.3	3.1-9.0	38.9	2.7	2.1-3.5	48.9	2.9	2.3-3.7	51.9	2.7	2.2-3.4
**55-64**	30.0	7.5	4.5-12.5	44.1	3.0	2.4-4.0	55.9	3.3	2.6-4.2	58.1	3.0	2.4-3.8
**65-74**	36.2	9.1	5.4-15.1	47.4	3.3	2.5-4.3	60.3	3.6	2.8-4.5	61.7	3.2	2.6-4.0
**75+**	31.8	8.0	4.7-13.5	33.1	2.3	1.7-3.1	52.1	3.1	2.4-4.0	56.8	2.9	2.3-3.7
**Female**												
	**WHO**			**NCEP-ATP III**			**IDF**			**JIS**		
**Age groups**	**%**	**PR**	**95% CI**	**%**	**PR**	**95% CI**	**%**	**PR**	**95% CI**	**%**	**PR**	**95% CI**
**25-34**	0.9	1.0		5.8	1.0		6.7	1.0		7.1	1.0	
**35-44**	2.6	2.9	1.0-8.8	13.0	2.2	1.5-3.5	17.8	2.7	1.8-4.0	17.8	2.5	1.7-3.7
**45-54**	8.8	9.8	3.6-26.9	27.5	4.7	3.3-7.3	32.4	4.8	3.4-7.2	32.8	4.6	3.3-6.7
**55-64**	14.4	16.0	5.9-43.2	38.7	6.7	4.6-10.2	45.2	6.8	4.8-9.9	46.2	6.5	4.7-9.4
**65-74**	23.4	26.0	9.7-69.6	47.6	8.2	5.7-12.5	57.7	8.6	6.2-12.6	59.0	8.3	6.0-11.9
**75+**	33.3	37.0	13.8-99.2	54.7	9.4	6.6-14.4	65.6	9.8	7.0-14.4	66.9	9.4	6.8-13.6

PR=prevalence ratio

### Prevalence of the components

All included definitions were composed of components indicating central obesity, lipid metabolism, blood pressure and glucose metabolism. For WHO, the criteria for triglycerides and HDL-C were combined to one lipid metabolism component and the total number of components was four. All other definitions included five components as triglycerides and HDL-C were individual components. To fulfil the criteria, at least three components had to exceed the defined thresholds for all definitions. In addition, in two definitions, one component was specified to be a prerequisite to fulfil their criteria [4, 27]. When looking at individual components of the MetS definitions, it was observed that the prevalence of different components varied between the definitions.

In persons/participants identified as having MetS, obesity, elevated blood pressure and elevated glucose levels/diabetes were present in over 80% of individuals by all four definitions. For WHO, glucose/diabetes and for IDF, obesity was the required component and therefore, all individuals defined as having MetS had these components for these definitions (Table 6). Among participants identified as having MetS, central obesity was the most prevalent component according to the IDF (prerequisite component) and JIS definitions, whereas NCEP-ATP recognized obesity, elevated blood pressure and glucose component practically as commonly. For WHO, the prerequisite component, glucose, was followed by the obesity component.

**Table 6:** Prevalence of components by MetS definition in participants identified as having MetS^a^

Component	WHO (1998) (*n*=1007)	95% CI	NCEP-ATP III (2004) (*n*=1896)	95% CI	IDF (2005) (*n*=2360)	95% CI	JIS (2009) (*n*=2448)	95% CI
Obesity	99.3%	98.6-99.7	86.6%	85.0-88.1	100.0%*	99.8-100.0	96.4%	95.6-97.1
Lipid metabolism^b^	51.6%	48.5-54.7						
Triglycerides			56.1%	53.9-58.3	46.1%	44.1-48.1	47.3%	45.3-49.3
HDL-C			40.1%	37.9-42.3	33.5%	31.6-35.4	33.8%	32.0-35.7
Blood pressure	90.6%	88.6-92.3	85.9%	84.3-87.4	91.4%	90.2-92.5	91.6%	90.4-92.6
Glucose/diabetes	100.0%*	99.6-100.0	84.7%	83.0-86.3	83.9%	82.4-85.3	84.2%	82.7-85.6

* Required component

^a^Prevalences were calculated in participants classified as having MetS, separately for each definition

^b^Lipid metabolism included both triglycerides and HDL-C in the WHO definition

The prevalence of the components in the total study population are presented in Additional files 2,3,4,5 for each definition. When examining the prevalence of the components by gender, obesity was the most prevalent component for both genders for the WHO definition (Additional file 2). For NCEP-ATP III, blood pressure was the most prevalent component for both genders, whereas the IDF and JIS criteria identified elevated blood pressure as the most common component for men and obesity for women (Additional files 3,4,5). Unlike the other definitions using only waist circumference, the WHO definition for obesity was based on waist-to-hip-ratio (cut-off points; m: 0.90, f: 0.85) and BMI (BMI≥ 30 kg/m^2^).The prevalence of these sub-components differed considerably being 87.5% and 25.4% for men, respectively, and 64.8% and 26.3% for women, respectively (Additional file 2).

## Discussion

The current study compared four different commonly used MetS definitions, the WHO [4], the NCEP-ATP III [28], the IDF [27] and the JIS [29] definitions. According to these definitions, MetS prevalence, calculated in the same study population in Finland, ranged considerably from 17.7 to 43.0%. Moreover, high discrepancy of participants identified by the different MetS definitions was seen. This emphasizes the need of using standardized definitions in order to make results comparable between countries and over time.

In our study, men had higher MetS prevalence than women according to all definitions. Central obesity and elevated blood pressure were the most common components in our study sample. This observation reflects the observed decline in blood glucose and cholesterol levels in the FinHealth 2017 survey compared to earlier Finnish surveys [36]. Interestingly, even though several European studies have identified distorted lipid metabolism as the most prevalent MetS component [25], in this study the most common risk factors were central obesity and elevated blood pressure in all age groups and both genders. However, our findings are coherent with a Spanish study for both genders and most age groups [23].

Overall, the differences between the definitions have been examined also previously among the European adult population with parallel findings on the WHO, NCEP-ATP III, IDF or JIS definitions [18, 19, 37, 38]. The definitions have evolved with time to be more encompassing such as including medication uptake and tighter cut-off points. Notably, IDF and JIS definitions [27, 29] also include ethnic specific cut-off points for waist circumference to enhance global comparability. Thus, when analyzing studies that apply the IDF or JIS criteria, it is also important to report which waist circumference cut-off points are used. MetS has been considered more prevalent among older population, and studies have traditionally tended to include only older participants. Our study especially wanted to include also the younger population to see how the different definitions and components manifest among this population. In our study, the focus was placed on explicit description and information on the definition criteria, as in many previous studies the details have not been clearly stated. Additionally, our study was based on relatively recent national population-based data among the Finnish population.

The WHO definition [4] has been proposed to be more suitable for academic settings due to its complexity and need for more specific biochemical examinations [39]. Furthermore, the less strict cut-off points of the WHO definition reflect the earlier treatment guidelines for non-communicable diseases [32, 40]. The NCEP-ATP III definition [28] resembles the more recent definitions of IDF and JIS [27, 29] and differs mainly in consideration of receiving treatment and less strict cut-off points for waist circumference. Notably, within previous studies, the definitions have often been modified [19, 30, 31]. As different versions of the definitions are used, it is of great importance that the included components and used cut-offs are always accurately described when results on MetS are presented.

As the MetS definitions require three or more components out of four or five components to fulfill the criteria, the identified subjects had different combinations of MetS components. In this study population, central obesity was highly prevalent among both men and women, similarly to other European populations [21, 23, 41]. However, the cut-off point for waist circumference was 8 cm higher for NCEP-ATP III compared to IDF and JIS, and thereby the prevalence of central obesity component was markedly lower using the NCEP-ATP III definition than that of IDF or JIS in this study (among men 37.7% and 63.4%, respectively, and among women 50.0% and 72.0%, respectively). The WHO central obesity component was based on two parts of which the prevalence of waist-to-hip ratio was much higher than that of BMI.

Overall, in our study as well as in previously published studies, using different MetS definitions resulted in quite different prevalence rates [18, 20]. Studies linking survey data with mortality follow-up data or data on the use of health care have been performed and they might be useful in evaluating the pros and cons of the MetS definitions [42, 43].

### Strengths and limitations

The results of this study represent the subjects who participated the FinHealth 2017 Survey and cannot be generalized to the whole Finnish population. However, they provide evidence on how greatly methodological choices may affect the results on the prevalence of MetS and thereby the interpretation of the study outcomes.

A strength of this study was the use of standardized procedures of the European Health Examination Surveys (EHES), providing harmonized protocols for national surveys and for European comparisons [34, 44]. Furthermore, our study population was based on a nationally representative random sample.

One of the limitations in our study was the short fasting time, only 4 h, required from the study participants before the blood sample draw. Most often fasting plasma glucose measurement includes a caloric restriction of 8 h as a prerequisite before the sampling [45]. However, in most field studies long fasting time is not feasible due to practicalities. In the FinHealth 2017 survey, minimum fasting time was set on four hours, which, however, has been suggested to be comparable with eight hours fasting for blood glucose measurements [46]. Differently from glucose profiles assessment, for assessing plasma lipids, short or no fasting time has been suggested to be comparable to longer fasting and should not be required before sample draw [47]. In population surveys, inconsistent fasting times and time of day of the sample draw may lead to variability in the results [48] and, consequently, bias the results. This variation is difficult to control. Another limitation of this study is that the WHO’s definition was modified to exclude microalbuminuria and HOMA-IR measurements due to lack of data on these issues in the FinHealth 2017 survey.

## Conclusions

The observed MetS prevalence had considerable variation depending on the used definition. The highest MetS prevalence was seen when using the JIS definition (43.0%) followed by IDF (41.5%), NCEP-ATP III (33.3%), and WHO (17.7%) definitions. The agreement for MetS prevalence was highest between the IDF and JIS definitions. The agreement was lowest for WHO vs. all other definitions.

Considering the public health burden and economic costs of MetS as well as the increasing global trend in the MetS prevalence, novel public health preventive actions are recommended. Regardless of the definition used, the prevalence of MetS among the younger participants in our study was high, hence preventive public health actions should be considered already among the younger populations. To ensure reliable research-based evidence to support public health actions and policy decisions, the use of standardized data collection methods and MetS definitions is essential. This implies a need for further discussion among MetS experts on the possibilities of selecting a “gold standard” definition.

## Supplementary information

**Additional file 1.**

**Additional file 2.**

**Additional file 3.**

**Additional file 4.**

**Additional file 5.**

## Data Availability

The FinHealth 2017 survey data are not publicly available due to restrictions based in the General Data Protection Regulation (GDPR) on sensitive data such as personal health data. The access to the data may be requested through the Finnish Institute for Health and Welfare (THL) Biobank (https://thl.fi/en/web/thl-biobank/for-researchers).

## Abbreviations

BMI: body mass index
CI: confidence interval
FPG: fasting plasma glucose
HDL-C: high-density lipoprotein cholesterol
HOMA-IR: Homeostatic Model Assessment of Insulin Resistance
IGT: impaired glucose tolerance
IDF: International Diabetes Federation
IFG: impaired fasting glucose
JIS: Joint Interim Statement
NCD: Non-communicable diseases
NCEP: National Cholesterol Education Program
NCEP-ATP III: National Cholesterol Education Program Adult Treatment Panel III
PR: prevalence ratio
T2DM: type 2 diabetes mellitus
TG: triglycerides
WC: waist circumference
WHO: World Health Organization
WHR: waist to hip ratio
