# Supplementary material for: Comparison of metabolic syndrome prevalence using four different definitions – a population-based study in Finland
Source: Arch Public Health. 2021 Dec 23;79:231. doi: 10.1186/s13690-021-00749-3 (PMC8697452; doi:10.1186/s13690-021-00749-3)

Comparison of metabolic syndrome prevalence using four different definitions – a population-based study in Finland

Haverinen E., Paalanen L., Palmieri L., Padron-Monedero A., Noguer-Zambrano I., Sarmiento Suárez R. & Tolonen, H.

Additional file 1.

A. Metabolic syndrome (MetS) prevalence (%) in men in different age groups

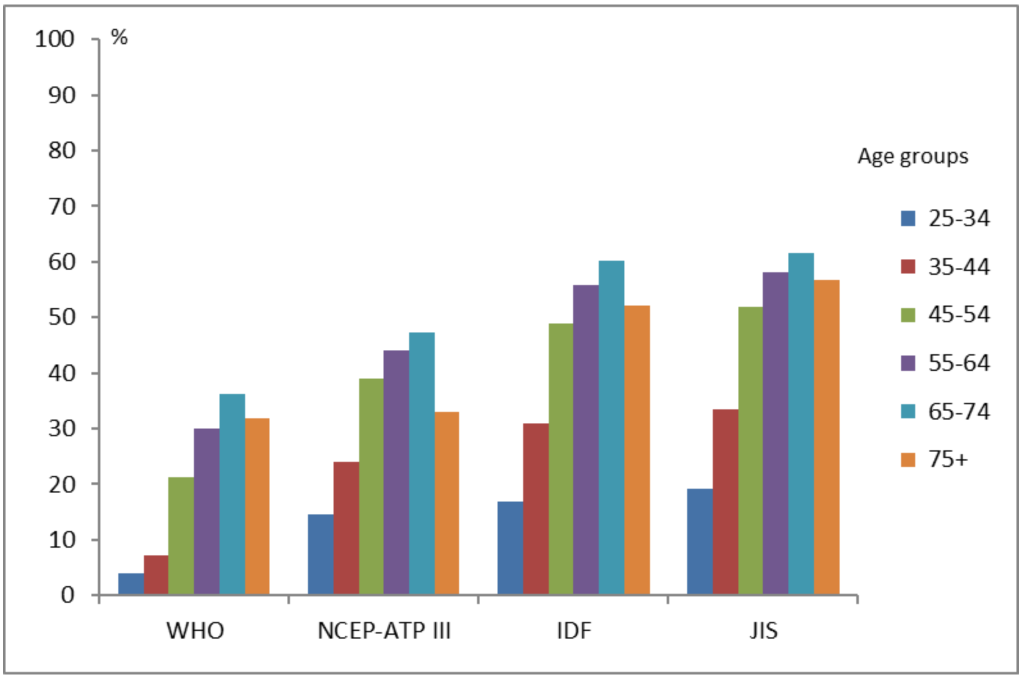

B. Metabolic syndrome (MetS) prevalence (%) among women in different age groups

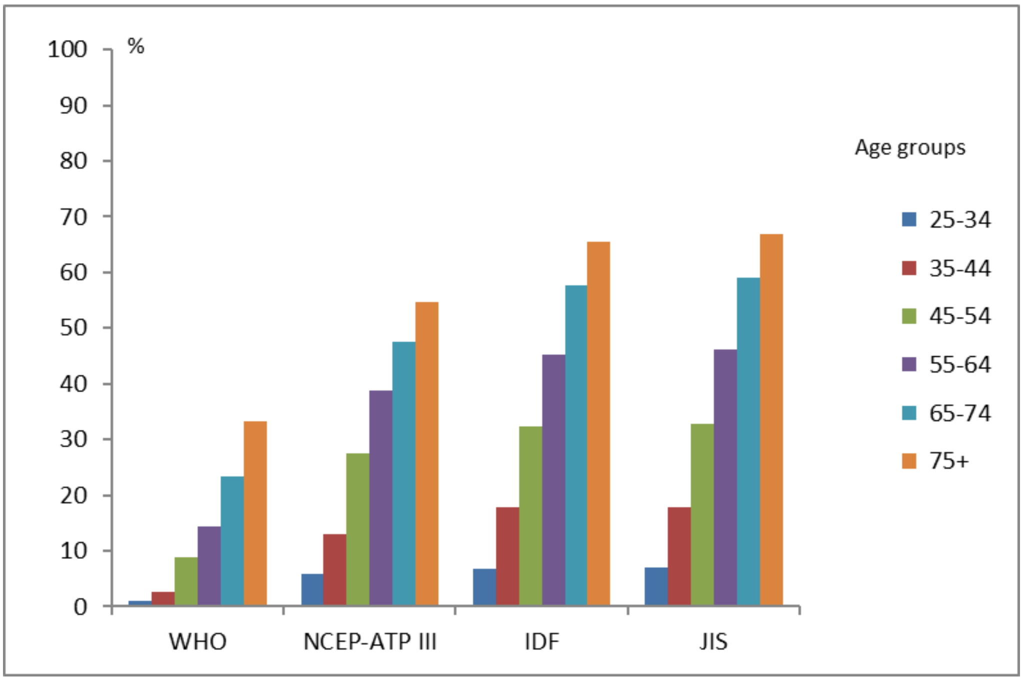

Supplement: Supplementary file 1 — Additional file 1. [file 13690_2021_749_MOESM1_ESM.pdf]
