# Supplementary material for: Comparison of metabolic syndrome prevalence using four different definitions – a population-based study in Finland
Source: Arch Public Health. 2021 Dec 23;79:231. doi: 10.1186/s13690-021-00749-3 (PMC8697452; doi:10.1186/s13690-021-00749-3)
Supplement: Supplementary file 2 — Additional file 2. [file 13690_2021_749_MOESM2_ESM.pdf]

Comparison of metabolic syndrome prevalence using four different definitions – a population-based study in Finland

Haverinen E., Paalanen L., Palmieri L., Padron-Monedero A., Noguer-Zambrano I., Sarmiento Suárez R. & Tolonen, H.

Additional file 2.

Prevalence of WHO metabolic syndrome components for total population, by gender and age groups

Total population and 95% CI identified with WHO obesity component (BMI ≥30 or waist-to-hip-ratio (WHR) m: 0.90, f: 0.85) (%)	Age groups	WHO obesity component, men (%)	95% CI	WHO obesity component, women (%)	95% CI
75.3 (74.2-76.4)	**Total**	87.5	86.2-88.7	64.8	63.1-66.5
	**25-34**	65.7	60.7-70.3	40.5	36.0-45.2
	**35-44**	80.8	76.8-84.3	53.6	49.2-58.0
	**45-54**	92.2	89.4-94.3	65.1	61.0-69.0
	**55-64**	93.0	90.7-94.8	72.9	69.3-76.3
	**65-74**	94.4	92.1-96.1	75.9	72.3-79.1
	**75-**	95.7	92.3-97.6	75.7	71.1-79.8
**BMI (%)**	**Age groups**	**WHO BMI, men (%)**	**95% CI**	**WHO BMI, women (%)**	**95% CI**
25.9 (24.8-27.1)	**Total**	25.4	23.8-27.1	26.3	24.8-27.9
	**25-34**	20.4	16.6-24.8	15.7	12.6-19.4
	**35-44**	20.8	17.2-24.9	21.0	17.6-24.8
	**45-54**	28.3	24.4-32.5	28.5	24.9-32.4
	**55-64**	30.9	27.3-34.7	29.1	25.7-32.8
	**65-74**	29.3	25.5-33.4	29.7	26.2-33.5
	**75-**	13.5	9.7-18.4	32.2	27.6-37.1
**WHR (%)**	**Age groups**	**WHO WHR, men (%)**		**WHO WHR, women (%)**	
72.6 (71.4-73.7)	**Total**	87.2	85.9-88.4	60.2	58.5-61.9
	**25-34**	64.6	59.6-69.3	35.3	31.0-39.9
	**35-44**	80.3	76.2-83.8	48.1	43.7-52.5
	**45-54**	91.8	89.0-93.9	59.9	55.7-63.9
	**55-64**	92.9	90.6-94.7	68.9	65.1-72.4
	**65-74**	94.4	92.1-96.1	71.8	68.1-75.2
	**75-**	95.3	91.8-97.3	72.6	67.8-76.9
**Total population and 95% CI identified with WHO lipid component (TG ≥ 150 mg/dl (1.7 mmol/L) and/ or M: HDL-C < 35 mg/dl (0.9 mmol/l) F: HDL-C < 39 mg/dl (1.0 mmol/l) (%)**	**Age groups**	**WHO lipid component, men (%)**	**95% CI**	**WHO lipid component, women (%)**	**95% CI**
24.2 (23.1-25.3)	**Total**	30.5	28.8-32.3	18.9	17.6-20.3
	**25-34**	20.1	16.3-24.5	7.4	5.3-10.3
	**35-44**	28.7	24.6-33.2	11.0	8.5-14.1
	**45-54**	43.1	38.7-47.6	19.0	15.9-22.5
	**55-64**	33.7	30.0-37.6	24.0	20.8-27.5
	**65-74**	28.7	25.0-32.7	24.2	21.0-27.8
	**75-**	20.8	16.1-26.4	25.2	21.0-29.918.3-33.6

**Total population and 95% CI identified with WHO blood pressure (BP) component (≥140/90 or medication) (%)**	**Age groups**	**WHO BP component, men (%)**	**95% CI**	**WHO BP component, women (%)**	**95% CI**
50.3 (49.0-51.6)	**Total**	54.5	52.6-56.4	46.8	45.0-48.6
	**25-34**	19.8	16.1-24.1	6.7	4.7-9.5
	**35-44**	29.8	25.6-34.3	14.6	11.8-18.0
	**45-54**	49.2	44.7-53.7	39.9	35.9-44.1
	**55-64**	68.1	64.3-71.7	54.6	50.7-58.5
	**65-74**	79.0	75.3-82.3	74.8	71.2-78.1
	**75-**	75.8	70.0-80.8	87.8	84.1-90.8
**Total population and 95% CI identified with WHO glucose component (FPG ≥ 110 mg/dl (6.1 mmol/l) or T2DM diagnosis) (%)**	**Age groups**	**WHO glucose component, men (%)**	**95% CI**	**WHO glucose component, women (%)**	**95% CI**
22.6 (21.5-23.7)	**Total**	28.3	26.6-30.1	17.8	16.5-19.2
	**25-34**	8.4	6.0-11.7	2.3	1.3-4.2
	**35-44**	15.0	11.9-18.7	6.1	4.3-8.6
	**45-54**	27.3	23.5-31.5	12.5	10.0-15.5
	**55-64**	34.2	30.5-38.1	19.2	16.3-22.5
	**65-74**	42.0	37.8-46.3	29.0	25.5-32.7
	**75-**	40.3	34.2-46.7	39.1	34.3-44.2

WHO= World Health Organization, BMI= body mass index, WHR= waist-to-hip-ratio, HDL-C= high density lipoprotein cholesterol, TGs= triglycerides, BP= blood pressure, T2DM= type 2 diabetes mellitus, FPG= fasting plasma glucose
