# Supplementary material for: Comparison of metabolic syndrome prevalence using four different definitions – a population-based study in Finland
Source: Arch Public Health. 2021 Dec 23;79:231. doi: 10.1186/s13690-021-00749-3 (PMC8697452; doi:10.1186/s13690-021-00749-3)
Supplement: Supplementary file 3 — Additional file 3. [file 13690_2021_749_MOESM3_ESM.pdf]

**Comparison of metabolic syndrome prevalence using four different definitions – a population-based study in Finland**

Haverinen E., Paalanen L., Palmieri L., Padron-Monedero A., Noguer-Zambrano I., Sarmiento Suárez R. & Tolonen, H.

Additional file 3.

Prevalence of NCEP-ATP III metabolic syndrome components for total population, by gender and age groups

| **Total population and 95% CI identified with NCEP-ATP III obesity component (waist circumference (WC) M: 102 cm, f: 88 cm) (%)** | **Age groups** | **NCEP-ATP III WC component, men (%)** | **95% CI** | **NCEP-ATP III WC component, women (%)** | **95% CI** |
|---|---|---|---|---|---|
| 44.3 (43.0-45.6) | **Total** | 37.7 | 35.9-39.6 | 50.0 | 48.2-51.8 |
| | **25-34** | 16.1 | 12.7-20.2 | 22.1 | 18.5-26.2 |
| | **35-44** | 24.9 | 21.0-29.2 | 38.3 | 34.1-42.7 |
| | **45-54** | 38.4 | 34.1-42.8 | 48.2 | 44.0-52.4 |
| | **55-64** | 49.0 | 45.0-53.0 | 57.7 | 53.8-61.5 |
| | **65-74** | 50.1 | 45.8-54.4 | 63.6 | 59.7-67.3 |
| | **75-** | 37.3 | 31.4-43.6 | 66.1 | 61.1-70.7 |
| **Total population and 95% CI identified with NCEP-ATP III triglyceride component (TG ≥ 150 mg/dl (1.7 mmol/L) (%)** | **Age groups** | **NCEP-ATP III triglyceride component, men (%)** | **95% CI** | **NCEP-ATP III triglyceride component, women (%)** | **95% CI** |
| 22.9 (21.8-24.0) | **Total** | 28.8 | 27.1-30.6 | 17.8 | 16.5-19.2 |
| | **25-34** | 18.0 | 14.4-22.2 | 6.5 | 4.5-9.2 |
| | **35-44** | 27.3 | 23.3-31.7 | 9.3 | 7.0-12.2 |
| | **45-54** | 42.0 | 37.6-46.5 | 17.8 | 14.8-21.2 |
| | **55-64** | 31.5 | 27.9-35.3 | 23.7 | 20.5-27.2 |
| | **65-74** | 27.2 | 23.5-31.2 | 22.9 | 19.7-26.4 |
| | **75-** | 18.6 | 14.2-24.1 | 24.4 | 20.3-29.0 |
| **Total population and 95% CI identified with NCEP-ATP III HDL-C component M: HDL-C < 40 mg/dl (1.03 mmol/l) F: HDL-C < 50 mg/dl (1.3 mmol/l) (%)** | **Age groups** | **NCEP-ATP III HDL-C component, men (%)** | **95% CI** | **NCEP-ATP III HDL-C component, women (%)** | **95% CI** |
| 17.8 (16.8-18.8) | **Total** | 15.5 | 14.2-16.9 | 19.8 | 18.4-21.2 |
| | **25-34** | 15.5 | 12.2-19.5 | 16.4 | 13.2-20.2 |
| | **35-44** | 16.4 | 13.2-20.2 | 22.5 | 19.0-26.4 |
| | **45-54** | 16.2 | 13.2-19.8 | 19.8 | 16.7-23.3 |
| | **55-64** | 14.9 | 12.3-18.0 | 17.2 | 14.4-20.4 |
| | **65-74** | 16.4 | 13.5-19.8 | 18.8 | 15.9-22.1 |
| | **75-** | 12.3 | 8.7-17.1 | 26.3 | 22.1-31.0 |
| **Total population and 95% CI identified with NCEP-ATP III blood pressure (BP) component (≥130/85) (%)** | **Age groups** | **NCEP-ATP III BP component, men (%)** | **95% CI** | **NCEP-ATP III BP component, women (%)** | **95% CI** |
| 60.0 (58.7-61.3) | **Total** | 66.0 | 64.2-67.8 | 54.9 | 53.1-56.7 |
| | **25-34** | 44.8 | 39.8-49.9 | 17.1 | 13.9-20.9 |
| | **35-44** | 53.9 | 49.1-58.6 | 28.4 | 24.6-32.5 |
| | **45-54** | 65.5 | 61.1-69.6 | 54.2 | 50.0-58.3 |
| | **55-64** | 74.0 | 70.3-77.4 | 64.0 | 60.1-67.7 |
| | **65-74** | 80.0 | 76.3-83.2 | 77.9 | 74.4-81.0 |
| | **75-** | 70.8 | 64.7-76.2 | 82.7 | 78.5-86.2 |

| **Total population and 95% CI identified with NCEP-ATP III glucose component (FPG >100 mg/dl (5.6 mmol/l) or medication) (%)** | **Age groups** | **NCEP-ATP III glucose component, men (%)** | **95% CI** | **NCEP-ATP III glucose component, women (%)** | **95% CI** |
|---|---|---|---|---|---|
| 50.4 (49.1-51.7) | **Total** | 60.1 | 58.2-62.0 | 42.1 | 40.4-43.9 |
| | **25-34** | 40.2 | 35.3-45.3 | 11.8 | 9.1-15.2 |
| | **35-44** | 47.3 | 42.6-52.1 | 19.9 | 16.6-23.7 |
| | **45-54** | 59.2 | 54.7-63.5 | 38.3 | 34.3-42.4 |
| | **55-64** | 65.9 | 62.9-69.6 | 47.5 | 43.6-51.4 |
| | **65-74** | 73.2 | 69.2-76.8 | 63.4 | 59.5-67.1 |
| | **75-** | 72.0 | 66.0-77.3 | 69.4 | 64.5-73.9 |

NCEP-ATP III= National Cholesterol Education Program Adult Treatment Panel III, WC= waist circumference, HDL-C= high density lipoprotein cholesterol, TGs= triglycerides, BP= blood pressure, T2DM= type 2 diabetes mellitus, FPG= fasting plasma glucose
