# Supplementary material for: Comparison of metabolic syndrome prevalence using four different definitions – a population-based study in Finland
Source: Arch Public Health. 2021 Dec 23;79:231. doi: 10.1186/s13690-021-00749-3 (PMC8697452; doi:10.1186/s13690-021-00749-3)
Supplement: Supplementary file 5 — Additional file 5. [file 13690_2021_749_MOESM5_ESM.pdf]

Comparison of metabolic syndrome prevalence using four different definitions – a population-based study in Finland

Haverinen E., Paalanen L., Palmieri L., Padron-Monedero A., Noguer-Zambrano I., Sarmiento Suárez R. & Tolonen, H.

Additional file 5.

Prevalence of JIS metabolic syndrome components for total population, by gender and age groups

**Total population and 95% CI identified with JIS obesity component (waist circumference (WC) M: 94 cm, F: 80 cm) (%)**	**Age groups**	**JIS WC component, men (%)**	**95% CI**	**JIS WC component, women (%)**	**95% CI**
68.1 (66.9-69.3)	**Total**	63.4	61.5-65.2	72.0	70.4-73.6
	**25-34**	32.2	27.7-37.1	45.9	41.3-50.6
	**35-44**	52.5	47.7-57.2	61.3	56.9-65.5
	**45-54**	64.7	60.3-68.9	72.2	68.3-75.8
	**55-64**	75.2	71.6-78.5	81.0	77.7-83.9
	**65-74**	75.9	72.0-79.4	82.4	79.2-85.2
	**75-**	72.9	66.9-78.2	84.8	80.8-88.1
**Total population and 95% CI identified with JIS triglyceride component (TG ≥ 150 mg/dl (1.7 mmol/L) (%)**	**Age groups**	**JIS triglyceride component, men (%)**	**95% CI**	**JIS triglyceride component, women (%)**	**95% CI**
22.9 (21.8-24.0)	**Total**	28.8	27.1-30.6	17.8	16.5-19.2
	**25-34**	18.0	14.4-22.2	6.5	4.5-9.2
	**35-44**	27.3	23.3-31.7	9.3	7.0-12.2
	**45-54**	42.0	37.6-46.5	17.8	14.8-21.2
	**55-64**	31.5	27.9-35.3	23.7	20.5-27.2
	**65-74**	27.2	23.5-31.2	22.9	19.7-26.4
	**75-**	18.6	14.2-24.1	24.4	20.3-29.0
**Total population and 95% CI identified with IDF HDL-C component M: HDL-C < 40 mg/dl (1.03 mmol/l) F: HDL-C < 50 mg/dl (1.3 mmol/l) (%)**	**Age groups**	**JIS HDL-C component, men (%)**	**95% CI**	**JIS HDL-C component, women (%)**	**95% CI**
17.8 (16.8-18.8)	**Total**	15.5	14.2-16.9	19.8	18.4-21.2
	**25-34**	15.5	12.2-19.5	16.4	13.2-20.2
	**35-44**	16.4	13.2-20.2	22.5	19.0-26.4
	**45-54**	16.2	13.2-19.8	19.8	16.7-23.3
	**55-64**	14.9	12.3-18.0	17.2	14.4-20.4
	**65-74**	16.4	13.5-19.8	18.8	15.9-22.1
	**75-**	12.3	8.7-17.1	26.3	22.1-31.0
**Total population and 95% CI identified with JIS blood pressure (BP) component (≥130/85 or medication) (%)**	**Age groups**	**JIS BP component, men (%)**	**95% CI**	**JIS BP component, women (%)**	**95% CI**
65.5 (64.3-66.7)	**Total**	71.8	70.0-73.5	60.0	58.3-61.7
	**25-34**	45.8	40.8-50.9	17.3	14.0-21.1
	**35-44**	54.6	49.8-59.3	29.4	25.6-33.6

	**45-54**	69.3	65.0-73.3	57.7	53.5-61.8
	**55-64**	81.4	78.1-84.3	71.0	67.3-74.4
	**65-74**	89.8	86.9-92.1	86.5	83.6-89.0
	**75-**	85.2	80.1-89.2	93.0	89.9-95.2
**Total population and 95% CI identified with JIS glucose component (FPG ≥ 100 mg/dl (5.6 mmol/l) or medication) (%)**	**Age groups**	**JIS glucose component, men (%)**	**95% CI**	**JIS glucose component, women (%)**	**95% CI**
50.4 (49.1-51.7)	**Total**	60.1	58.2-62.0	42.1	40.4-43.9
	**25-34**	40.2	35.3-45.3	11.8	9.1-15.2
	**35-44**	47.3	42.6-52.1	19.9	16.6-23.7
	**45-54**	59.2	54.7-63.5	38.3	34.3-42.4
	**55-64**	65.9	62.9-69.6	47.5	43.6-51.4
	**65-74**	73.2	69.2-76.8	63.4	59.5-67.1
	**75-**	72.0	66.0-77.3	69.4	64.5-73.9

JIS= Joint Interim Statement, WC= waist circumference, HDL-C= high density lipoprotein cholesterol, TGs= triglycerides, BP= blood pressure, FPG= fasting plasma glucose
